# Magnitude and determinants of nonadherence and nonreadiness to highly active antiretroviral therapy among people living with HIV/AIDS in Northwest Ethiopia: a cross - sectional study

**DOI:** 10.1186/1742-6405-7-2

**Published:** 2010-01-14

**Authors:** Belay Tessema, Fantahun Biadglegne, Andargachew Mulu, Assefa Getachew, Frank Emmrich, Ulrich Sack

**Affiliations:** 1 Department of Medical Laboratory Technology, College of Medicine and Health Sciences, University of Gondar, Gondar, Ethiopia; 2 Department of Microbiology, Immunology and Parasitology, College of Medicine and Health Sciences, University of Bahir Dar, Bahir Dar, Ethiopia; 3 Department of Microbiology and Parasitology, College of Medicine and Health Sciences, University of Gondar, Gondar, Ethiopia; 4 Department of Radiology, College of Medicine and Health Sciences, University of Gondar, Gondar, Ethiopia; 5 Institute of Clinical Immunology, Faculty of Medicine, University of Leipzig, Leipzig, Germany; 6 Institute of Medical Microbiology and Epidemiology of Infectious diseases, Faculty of Medicine, University of Leipzig, Leipzig, Germany; 7 Fraunhofer Institute for Cell Therapy and Immunology, Leipzig, Germany

## Abstract

**Background:**

Adequate antiretroviral drug potency is essential for obtaining therapeutic benefit, however, the behavioral aspects of proper adherence and readiness to medication, often determine therapeutic outcome. Therefore, this study aimed to assess the level and determinants of nonadherence and nonreadiness to highly active antiretroviral therapy (HAART) among people living with HIV/AIDS (PLWHA) at Gondar University Teaching Hospital and Felege Hiwot Hospital in Northwest Ethiopia.

**Methods:**

A cross-sectional study was conducted between July and September 2008 using structured interviewer-administered questionnaire. All consecutive adult outpatients who were receiving antiretroviral treatment for at least three months, seen at both hospitals during the study period and able to give informed consent were included in the study. Multivariate logistic regression was used to determine factors associated with nonadherence and nonreadiness.

**Results:**

A total of 504 study subjects were included in this study. The prevalence rates of nonadherence and nonreadiness to HAART were 87 (17.3%) and 70 (13.9%) respectively. Multivariate logistic regression analysis revealed that medication adverse effects, nonreadiness to HAART, contact with psychiatric care service and having no goal had statistically significant association with nonadherence. Moreover, unwillingness to disclose HIV status was significantly associated with nonreadiness to HAART.

**Conclusions:**

In this study the level of nonadherence and nonreadiness to HAART seems to be encouraging. Several factors associated with nonadherance and nonreadiness to HAART were identified. Efforts to minimize nonadherence and nonreadiness to HAART should be integrated in to regular clinical follow up of patients.

## Introduction

HIV/AIDS is the fourth most common cause of death in the world [1] and is estimated to have killed 3.1 million individuals and infected 4.9 million persons in 2005 alone. The number of people infected by HIV is steadily rising and sub-Saharan Africa is the most affected region in the world [2]. Ethiopia has the fifth largest population of HIV-infected individuals living in Africa, which accounts approximately 4% of the world's HIV/AIDS cases [3].

Highly Active Antiretroviral Treatment (HAART) has dramatically reduced mortality and morbidity due to HIV [4,5]. It is effective because it reduces HIV replication and hence allows the regeneration of CD4+ T-lymphocyte mediated immune responses [6,7]. It cannot, however, totally eradicate HIV [8,9] and hence prolonged viral suppression is essential for long-term efficacy of HAART [10,11].

Prolonged viral suppression is only achievable if the virus does not get the chance to replicate and develop drug-resistant HIV variants [12]. The virus has the chance to replicate not only if the patient is untreated [13] but also if the viral replication is not completely inhibited by the treatment (i.e. due to sub-optimal drug exposure) [14]. When replication occurs during treatment, this leads to the development of genetic variation, which in turn leads to the emergence of variants that might be resistant to antiretroviral treatment [12].

Despite the high prevalence of HIV/AIDS in Africa including Ethiopia, the HAART coverage is extremely low due to limited resources, but in these days WHO as well as different countries are interested to intensify the HAART activities and expand the program as preventive strategy for HIV epidemic and AIDS patient care[15].

Ethiopia has been started provision of HAART for the people living with HIV/AIDS since August 2003. However, by the end of June 2008, there were only 110,611 patients (75%) who were alive and on HAART out of the 150,136 patients who had been started on HAART since 2003 [16]. This indicates the need for an intervention to reduce the drop-out rate due to either death or loss to follow-up.

One of the main factors contributing to sub-optimal drug levels and resistance is non-adherence to treatment [17,18]. It has been reported that the patient needs to take a minimum of 95% of prescribed antiretroviral doses in order to avoid resistance development. Patients taking 95% or more of their doses only had a documented virologic failure (i.e. over 400 virus copies/mL in blood) in 22% of the cases compared to 80% of the patients taking less than 80% of their doses [17].

Patient's readiness to antiretroviral therapy means put the patient himself/herself feels ready to initiate, take responsibility for, and to maintain (including being adherent to) a prescribed treatment [19]. Readiness for treatment can be assessed prior to treatment initiation and hence timely measures can be taken before initiation of therapy, sometimes postponement of treatment may be preferable in order to motivate and increase the degree of readiness, and hence, hopefully, increase the success rate of the treatment [20].

Assessment of patient adherence and readiness to treatment are good opportunities to enhance patient understanding of medication regimen, to identify potential obstacles to taking medication and trusting relationship between patients and health care providers, and ultimately to prevent virologic break through [21]. Therefore, this study aimed to assess the level and determinants of nonadherence and nonreadiness to HAART among PLWHA at Gondar University Teaching Hospital and Felege Hiwot Hospital in Northwest Ethiopia.

## Methods

### Study design, area, and period

A cross-sectional study was conducted between July and September 2008 at Gondar University Teaching Hospital and Felege Hiwot Hospital in Northwest Ethiopia. These hospitals are tertiary level teaching hospitals that each hospital provides health service to over five million inhabitants in Northwest Ethiopia, located 727 Km and 540 Km away from the capital city, Addis Ababa respectively.

### Study subjects

All consecutive adult outpatients who were receiving antiretroviral treatment at least for three months, seen at both clinics during the study period and able to give informed consent were included in the study.

### Data collection

Data was collected using structured interviewer-administered questionnaires which include the following variables: socio-demographic characteristics, knowledge of patients towards treatment and health care system, patient attitude towards health care provider and program and patients self-report to treatment adherence and readiness. Prior to data collection, training to nurses (interviewers) about the objectives of the study and methods of interviewing was given and the English version questionnaire was translated to the local language (Amharic). Institutional ethical clearance was obtained from the research and publication committee of Gondar University.

### Assessments

#### Adherence

Respondents were asked whether they had missed any doses the day prior to completing the questionnaire, and how often doses were missed in general (ranging from every day to never). Respondents, who were reported that they had not forgotten a dose the day prior to the completion of the questionnaire and those responded that they never forget doses were categorized as adherent. This strict definition of adherence was chosen, since the respondents providing us with these answers would theoretically reach an adherence level of at least 95% [17].

#### Readiness

Patients' readiness to HAART was determined based on the five indicators of readiness [19]: These indicators are: Changing attitudes towards HIV medication, finding the right health care provider, creating the right support system, getting control over life and having goals. Changing attitudes towards HIV medication was assessed by asking the patients if they thought that their present treatment would prevent them from becoming ill as a result of their HIV infection. Finding the right health care provider was assessed by asking the patients how they perceive their contacts with health care staffs. Creating the right support system was measured by two-question assessment of social support for taking medicine. First patients were asked whether they had friends or relatives to talk about their treatment, second patients were asked whether they had friends or relatives who reminded them to take their medicine. Patients answering no to both of these items were considered as lacking social support. Getting control over life was assessed by asking whether the patient has a special system to remember the medication. The last indicator, having goals was assessed by asking whether the patient is developing and maintaining specific goals when living with HIV. Goals could be relatively long-term, intermediate, or even very short-term. What's important is maintaining incentives to live, to feel that there's something left to accomplish or to learn or to contribute to the world. Respondents, who presented the aforementioned five indicators of readiness, were categorized as ready to HAART.

### Statistical analysis

Data was coded, cleared, entered and analysed using SPSS statistical software version 13. Different variables were described and characterized by frequency distribution. Association between the dependent and independent variables was analyzed using chi- square test and multivariate logistic regression. In all cases p-value of less than 0.05 was taken to indicate level of statistical significance.

## Results

### Socio-demographic characteristics

A total of 504 study subjects (252 from each hospital) were included in this study. Of these, 310 (61.5%) respondents were females and 194 (38.5%) were males with the mean (SD) age of 35.3 (8.9) years. Most of the study subjects, 452 (89.7%) were urban resident, 488 (96.8%) were Amhara by ethnicity and 471 (93.5%) were Christian by religion. Large number of the respondents, 138 (27.4%) were illiterate, 107 (21.2%) were widowed, 114 (22.6%) were daily labourers and 254 (50.4%) had household income below 45.25 USD per month. Moreover, thirty six (7.1%) study subjects were reported active substance use (alcohol, Khat and/or cigarette) (Table 1).

**Table 1:** Socio-demographic characteristics of PLWHA, Felege Hiwot Hospital and Gondar University Teaching Hospital, Northwest Ethiopia, 2008

Socio demographic characteristics	Felege Hiwot Hospital		Gondar University Hospital		Total	
	
	Number	Percent	Number	Percent	Number	Percent
**Sex**						
Male	98	38.9	96	38.1	194	38.5
Female	154	61.1	156	61.9	310	61.5
**Age group (Years)**						
18 - 24	11	4.4	28	11.1	39	7.7
25 - 34	95	37.7	102	40.5	197	39.1
35 - 44	109	43.3	83	32.9	192	38.1
45 - 54	28	11.1	33	13.1	61	12.1
≥55	9	3.6	6	2.4	15	3.0
**Address**						
Urban	217	86.1	235	93.3	452	89.7
Rural	35	13.9	17	6.7	52	10.3
**Educational status**						
Illiterate	70	27.8	68	27.0	138	27.4
Read and write	20	7.9	21	8.3	41	8.1
Primary school	77	30.6	44	17.5	121	24.0
Secondary school	65	25.8	92	36.5	157	31.2
Diploma and above	20	7.9	27	10.7	47	9.3
**Marital status**						
Single	27	10.7	47	18.7	74	14.7
Married	113	44.8	111	44.0	224	44.4
Divorced	47	18.7	52	20.6	99	19.6
Widowed	65	25.8	42	16.7	107	21.2
**Monthly income (USD)**						
< 45.25	113	44.8	141	56.0	254	50.4
45.25 - 90.41	3	1.2	6	2.4	9	1.8
> 90.41	79	31.3	53	21.0	132	26.2
Unstated	57	22.6	52	20.6	109	21.6
**Religion**						
Christian	229	90.9	242	96.0	471	93.5
Muslim	23	9.1	10	4.0	33	6.5
**Ethnicity**						
Amhara	246	97.6	242	96.0	488	96.8
Tigre	6	2.4	3	1.2	9	1.8
Oromo	-	-	2	0.8	2	0.4
Others	-	-	5	2.0	5	1.0
**Occupation**						
Merchant	35	13.9	38	15.1	73	14.5
Farmer	18	7.1	16	6.3	34	6.7
Student	5	2.0	12	4.8	17	3.4
Government employee	48	19.0	42	16.7	90	17.9
Daily labourer	56	22.2	58	23.0	114	22.6
NGO employee	25	9.9	25	9.9	50	9.9
Others	65	25.8	61	24.2	126	25.0
**Substance use**						
Yes	28	11.1	8	3.2	36	7.1
No	224	88.9	244	96.8	468	92.9

NGO = Non governmental organization

### Treatment, clinical, psychosocial and health service related variables

The study subjects were on HAART for a mean and median duration of 18.9 and 16.5 months respectively. Of all study subjects, 430 (85.3%) had disclosed their sero-status (to family members, friends and/or neighbors). Four hundred ninety five (98.2%) respondents thought that HAART had benefited them by improving their quality of life or improving their symptoms. One hundred ninety nine (39.5%) had an adverse reaction to HAART like skin rash, itching, nausea, and/or vomiting since starting HAART. More than half, 255 (50.6%) participants had discomfort when taking their drugs in front of others, and most of the respondents, 476 (94.4%) were satisfied with the health care providers service. Majority of respondents, 453 (89.9%) had access to pharmacy at any time and 108 (21.4%) patients were visiting their doctors monthly. Moreover, 59 (11.7%) respondents reported contact with psychiatric care services and 485 (96.2%) were having goals (Table 2).

**Table 2:** Treatment, psychosocial and health service related variables of PLWHA, Felege Hiwot Hospital and Gondar University Teaching Hospital, Northwest Ethiopia, 2008

Clinical and Psychosocial variables	Felege Hiwot Hospital		Gondar University Hospital		Total	
	
	Number	Percent	Number	Percent	Number	Percent
**Duration of treatment (months)**						
3 - 6	34	13.5	69	27.4	103	20.4
7 - 12	47	18.7	68	27.0	115	22.8
13 -18	31	12.3	22	8.7	53	10.5
19 - 24	47	18.7	49	19.4	96	19.0
≥ 25	93	36.9	44	17.5	137	27.2
**HIV status disclosure**						
Yes	230	91.3	200	79.4	430	85.3
No	22	8.7	52	20.6	74	14.7
**Treatment side effects**						
Yes	61	24.2	138	54.8	199	39.5
No	191	75.8	114	45.2	305	60.5
**Clinical follow up**						
Monthly	13	5.2	95	37.7	108	21.4
Every two months	8	3.2	91	36.1	99	19.6
Every three months	91	36.1	44	17.5	135	26.8
Variable	140	55.6	22	8.7	162	32.1
**Perceived access to Pharmacy**						
Yes	248	98.4	205	81.3	453	89.9
No	-	-	17	6.7	17	3.4
Not sure	4	1.6	30	11.9	34	6.7
**Belief on HAART benefits**						
Yes	250	99.2	245	97.2	495	98.2
No	-	-	5	2.0	5	1.0
I doubt	2	0.8	2	0.8	4	0.8
**Contact with psychiatric care services**						
Yes	16	6.3	43	17.1	59	11.7
No	236	93.7	209	82.9	445	88.3
**Perceived satisfaction with HCP**						
Yes	242	96.0	234	92.9	476	94.4
No	-	-	13	5.2	13	2.6
Not sure	10	4.0	5	2.0	15	3.0
**Having goals**						
Yes	249	98.8	236	93.7	485	96.2
No	3	1.2	16	6.3	19	3.8
**Comfortable when taking HAART in front of others**						
Yes	124	49.2	125	49.6	249	49.4
No	128	50.8	127	50.4	255	50.6

HCP = Health Care Providers

### Self reported nonadherence and nonreadiness to HAART among the study subjects

The level of nonadherence and nonreadiness to HAART were 87 (17.3%) and 70 (13.9%) respectively. The main reasons for nonadherence are drug side effects 27 (31.0%) and other health problems 19 (21.8%). On the other hand, the major reasons for nonreadiness to HAART are anxiety 31 (44.3%) and hopelessness 19 (27.1%). Of all study subjects, 419 (83.1%) were highly motivated to take HAART, 74 (14.7%) were partially motivated and 11 (2.2%) respondents were not at all motivated to take HAART (Table 3).

**Table 3:** Self reported nonadherence and nonreadiness to HAART, Felege Hiwot Hospital and Gondar University Teaching Hospital, Northwest Ethiopia, 2008

Variables	Felege Hiwot Hospital		Gondar University Hospital		Total	
	
	Number	Percent	Number	Percent	Number	Percent
**Adherence status**						
Adherent	231	91.7	186	73.8	417	82.7
Nonadherent	21	8.3	66	26.2	87	17.3
**Readiness status**						
Ready	242	96.0	192	76.2	434	86.1
Not ready	10	4.0	60	23.8	70	13.9
**Motivation to take HAART**						
Not at all motivated	1	0.4	10	4.0	11	2.2
Partially motivated	18	7.1	56	22.2	74	14.7
Highly motivated	233	92.5	186	73.8	419	83.1
**Reasons for nonadherence**						
Drug side effects	4	19.1	23	34.9	27	31.0
Other health problems	6	28.6	13	19.7	19	21.8
Getting a relief	1	4.8	4	6.1	5	5.75
Drug scarcity	2	9.5	2	3.0	4	4.6
Others	8	38.1	24	36.4	32	36.8
**Reasons for nonreadiness to HAART**						
Anxiety	-	-	31	51.7	31	44.3
Hopelessness	8	80.0	11	18.3	19	27.1
Confusion	-	-	14	23.3	14	20.0
Denial	2	10.0	1	1.7	3	4.3
Others	-	-	3	5.0	3	4.3

### Determinants of patients' nonadherence and nonreadiness to HAART

Results of multivariate logistic regression analyses showed that treatment adverse effects (P = 0.04; OR = 1.4; 95% CI = 0.8 - 2.5), nonreadiness to HAART (P < 0.001; OR = 8.9; 95% CI = 4.8 - 16.7), contact with psychiatric care service (P = 0.02; OR = 2.2; 95% CI = 1.1 - 4.5) and having no goal (P = 0.03; OR = 3.5; 95% CI = 1.1 - 10.8) had statistically significant association with nonadherence (Table 4). Moreover, unwillingness to disclose HIV status (P = 0.04; OR = 1.9; 95% CI = 1.1 - 3.5) was significantly associated with nonreadiness to antiretroviral therapy (Table 5).

**Table 4:** Association of variables with nonadherence to HAART, Felege Hiwot Hospital and Gondar University Teaching Hospital, Northwest Ethiopia, 2008

Determinants	Adherence status		Adjusted OR**	95% CI		P-Values
					
	Nonadherent N (%)	Adherent N (%)		Lower	Upper	
**Treatment side effects**						
No*	34 (39.1)	271 (65.0)	1.0	--	--	--
Yes	53 (60.9)	146 (35.0)	1.4	0.8	2.5	0.04
**Readiness to HAART**						
Ready*	46 (52.9)	388 (93.0)	1.0	--	--	--
Not ready	41 (47.1)	29 (7.0)	8.9	4.8	16.7	<0.001
**Contact with psychiatric care services**						
No*	69 (79.3)	376 (90.2)	1.0	--	--	--
Yes	18 (20.7)	41 (9.8)	2.2	1.1	4.5	0.02
**Having goals**						
Yes*	76 (87.4)	409 (98.1)	1.0	--	--	--
No	11 (12.6)	8 (1.9)	3.5	1.1	10.8	0.03

N = Number, OR = Odds ratio, CI = Confidence interval; * = Reference Category; ** = All the variables in the table are included in the model

**Table 5:** Association of variables with nonreadiness to HAART, Felege Hiwot Hospital and Gondar University Teaching Hospital, Northwest Ethiopia, 2008

Variables	Readiness status		Adjusted OR**	95% CI		P-Values
					
	Not ready N (%)	Ready N (%)		Lower	Upper	
**Sex**						
Male*	25 (35.7)	169 (38.9)	1.0	--	--	--
Female	45 (64.3)	265 (61.1)	1.1	0.7	1.9	0.636
**HIV status disclosure**						
Yes*	55 (78.6)	375 (86.4)	1.0	--	--	--
No	15 (21.4)	58 (13.6)	1.9	1.1	3.5	0.04
**Contact with psychiatric care services**						
No*	58 (82.9)	387 (89.2)	1.0	--	--	--
Yes	12 (17.1)	47 (10.8)	1.8	0.9	3.6	0.101

N = Number; OR = Odds ratio; CI = Confidence interval; * = Reference category; ** = All the variables in the table are included in the model

## Discussion

The prevalence of nonadherence and nonreadiness to HAART and their determinants among patients attending the antiretroviral clinics in Gondar and Felege Hiwot Hospitals in Northwest Ethiopia were the focuses of this study. Of all study subjects, 87 (17.3%) respondents had less than 95% adherence and 70 (13.9%) of the respondents had not been ready to HAART. The level of nonadherence in this study was comparable with those reported in Addis Ababa (capital city of Ethiopia) where adherence rates were 81.2% [22] and 82.8% [23], but it was lower than in most developed countries, where adherence rates ranged from 50% to 70% [24,25]. The low level of nonadherence in our study compared to in most developed countries might be due to the infancy stage of HAART program in the study areas.

The multivariate logistic regression analyses showed that medication adverse effects had statistically significant association with nonadherence to antiretroviral therapy. This is in agreement with the findings of other studies conducted in Brazil, Senegal and Addis Ababa [22,26,27]. Efforts to improve the level of adherence should be made by letting patients know at the start of the treatment which side effects are possible with a given regimen, monitoring for such effects and provide treatment for adverse effects even beginning with the first prescription.

Although there are few published studies, and they have used different methods to assess readiness, a significant association between the level of readiness and the level of adherence has been observed [28]. This observation is consistent with the finding of the current study. Contact with psychiatric care service also showed significant association with nonadherence to HAART. This is in agreement with studies conducted elsewhere [29-31]. This significant association might be due to the fact that most people with HIV, at some time in the course of their illness, experience a psychiatric disorder [32,33], and AIDS related dementia (AIDS Dementia Complex - ADC) characterized by abnormalities in cognitive as well as motor function [34].

Having long-term plans and goals, using time wisely and having a meaningful life are characteristics of patients who have fewer adherence difficulties [30,35]. This is also reflected in our study, that having no goal had significant association with nonadherence. Moreover, in the present study, unwillingness to disclose HIV status was significantly associated with nonreadiness to antiretroviral therapy (Table 5). This finding is consistent with the finding of other study where disclosure [36] is considered as barrier that prevent patients from wanting to start and to adhere to HAART.

Our study has the following limitations. First, we measured adherence and readiness of patients to HAART by patient self - report, which may be subject to recall bias and overestimate adherence and readiness. Nevertheless, many other studies document that well collected self report data clearly correlates with virologic changes and is more practical in most settings [37,38]. Second, we were unable to relate the obtained adherence rate to viral loads, CD4+ T-cell counts and clinical progression due to financial and logistical constraints. Comparison of reported adherence levels with viral loads, CD4+ T-cell counts and clinical progression would be beneficial in providing a more comprehensive view of adherence to HAART.

## Conclusions

In this study the level of nonadherence and nonreadiness to HAART seems to be encouraging. Medication adverse effects, nonreadiness to HAART, contact with psychiatric care service and having no goal were significant barriers to treatment adherence. Moreover, unwillingness to disclose HIV status was a significant factor for nonreadiness to HAART. Therefore, efforts to minimize nonadherence and nonreadiness to HAART should address these barriers among others, and should be integrated in to regular clinical follow up of patients. Furthermore, continuous measurement of patients' non adherence and nonreadiness, to identify when interventions are required, seems to be an approach worth further investigation.

## Competing interests

The authors declare that they have no competing interests.

## Authors' contributions

BT was the primary researcher, conceived the study, designed, conducted data analysis and drafted the manuscript for publication.

FB, AM and AG assisted in data collection and reviewed the initial and final drafts of the manuscript.

FE and US interpreted the results, and reviewed the initial and final drafts of the manuscript.

All the authors read and approved the final manuscript for submission for publication.
